# Swiss medical schools’ experiences with online teaching during the COVID-19 pandemic in light of international experiences

**DOI:** 10.1186/s12909-024-05218-3

**Published:** 2024-03-06

**Authors:** Artemisa Gogollari, Sharon Mitchell, Sissel Guttormsen

**Affiliations:** 1 Institute of Medical Education, University of Bern, Mittelstrasse 43, 3012 Bern, Switzerland; 2 Graduate School for Health Sciences, University of Bern, Bern, Mittelstrasse 43, 3012 Switzerland

**Keywords:** Medical education, COVID-19 pandemic, Digital learning and teaching

## Abstract

**Background:**

During the pandemic, all universities had to switch to digital learning and teaching (DLT), the experiences were diverse. The advantages and obstacles of DLT are well reported in research. To ensure a sustainable DLT implementation, the requirements of institutions, educators and students should be aligned.

**Objective:**

This paper aims at identifying and describing the experiences made at the Swiss medical schools after having to switch from on-site to on-line teaching; in particular, the experienced issues, requirements, and solutions were investigated and compared to international literature.

**Methods:**

We conducted a literature review to derive themes and subthemes regarding the central aspects of the transition from on-site to on-line teaching. Also, we conducted semi-structured interviews with people responsible for the medical curricula at the Swiss Medical Schools. We used a purposive sampling method and invited eleven curriculum managers at the seven Swiss Medical Schools. The interviews were conducted in English, audio-recorded and transcribed. Subsequently the data was analysed with the software NVivo. We used a qualitative, deductive, content analysis to explore faculty experiences.

**Results:**

Twenty-four articles met the eligibility criteria and were included for full text screening. Of the included articles, 15 reported on DLT in general and nine articles reported on DLT during the Pandemic. The thematic analysis of the interviews resulted in four overall themes, requirements, obstacles, facilitators and advantages. Curriculum managers reported that institutions were relatively unprepared for the quick transition from onsite to online at the onset of the pandemic.

**Conclusions:**

Our research reports a lack of institutional structures, communication, digital competences and literacy, teaching strategies, as well as a theoretical foundation for DLT implementation. A conceptual framework for DLT adapted to the Swiss universities beyond the current situation is needed.

**Supplementary Information:**

The online version contains supplementary material available at 10.1186/s12909-024-05218-3.

## Introduction

The Covid-19 pandemic (hereafter pandemic) had significant implications on education systems as it forced all universities worldwide to switch to online teaching. This shift towards ‘digital Learning and teaching’ (DLT), as a response to the crisis, seems to meet students’ preferences for flexible learning opportunities. Before the pandemic, students’ presence in onsite events was decreasing across all educational systems [1], still, the rate of implementation of DLT by Universities worldwide, and in Switzerland, remained slow [2–5]. In the context of this article, DLT refers to the utilization of electronic technologies and resources to facilitate the acquisition, development, and application of knowledge, skills, and attitudes within educational settings. Within this term we encompass both the broader, established approach of "online teaching and learning" and also "Emergency remote teaching" a rapid instructional mode implemented in response to sudden disruptions like the Corona Pandemic [6]. If implemented well, DLT has the potential to increase student engagement, support active learning and interaction with the learning material [7, 8] and enable students to personalize their learning [9]. Furthermore, DLT fosters peer learning and self-directed learning skills [10, 11]. In medical education, advantages of DLT include improved information accessibility, facilitation of standardised content, cost-effectiveness and accountability [12–14]. Obstacles for DLT implementation are also widely reported including a lack of institutional DLT structures, low awareness of benefits, and missing strategies for DLT implementation [5, 15]. Teachers acknowledge the values of DLT [16], but due to lack of institutional support [5], technical knowledge and time constraints [17], implementation of interactive online learning is lagging [18]. Presented solutions to these challenges are not always optimal [19]. For successful DLT implementation the requirements of all the key-players, students, teachers and institutions, must be addressed specifically.

Students were among the first to notice the consequences of the rapid change to online teaching. Their perceptions about online learning during the pandemic show a high degree of dissatisfaction [20–23]. While students report higher ‘learner control’ [24], they also report lack of additional instructional support and difficulty in adjusting their learning approach to DLT [25]. Reduced interaction and feedback with peers and educators are noted disadvantages of online learning [20, 21, 25]. The European Medical Student Association (EMSA) surveyed 11 countries on experiences during the pandemic [21], highlighting a low level of student involvement in decision-making, absence of a feedback mechanism and lack of quality resources. Similar data is reported in many Asian countries [20, 22, 23] and, in two comprehensive global reviews [26, 27].

Teachers need a new set of skills in order to support a digital teaching programme, such as communicating online, time management, technical literacy, and adapting to a new role as a facilitator [20, 28]. Effective DLT is not restricted to telling students what is known, but rather to scaffold learning. Published studies report that teachers are not aware of this role [29–31].

Also, educational institutions, particularly curriculum managers play a key-role in facilitating the requirements of teachers and students. Institutions have the decision power to implement a pedagogical framework, to provide an educational setting supporting DLT [32], to allocate resources [32, 33], and ensure effective implementation of DLT. A lack of guidance on the institutional level is often among the biggest obstacles to meaningful change [34]. While the pandemic resulted in an incredible effort to succeed in the delivery of effective online teaching, there is a risk that this spirit will fade away when the pandemic crisis is resolved [35]. To ensure that institutions and educators continue to maximise teaching and learning using both online and onsite, fitting to education needs of students, the current innovation momentum is essential to work towards optimal and sustainable DLT implementation, both didactically and organizationally [36]. The following research questions address crucial insights needed in this process:

- RQ 1: Which factors, known from research before the pandemic, are particularly relevant for DLT implementation in higher medical education?
- RQ 2 What is known from the literature about DLT experiences in medical education during the pandemic?
- RQ 3: Which DLT experiences do Swiss medical curriculum manager report from the pandemic and how do these experiences relate to the literature?

## Method

We applied two methodological approaches, a literature review and structured interviews, converging to an integrated analysis. We identified a set of themes and sub-themes to illustrate the transition from onsite to online teaching, which will be addressed below.

### Literature review

To address the first two research questions, we conducted a ‘literature review’ following Booth and Grant, 2009 [37], which is inherently semi-structured. We applied the following approach: 1) search for relevant studies (before and after the pandemic); 2) select appropriate studies based on pre-defined inclusion criteria; 3) extract and code data using NVivo; and 4) summarise the results using descriptive themes. The search code and the PRISMA flow diagram are shown in Supplementary Material 1: Appendix I.

In the first step, we reviewed evidence to contextualise knowledge in terms of existing DLT themes such as requirements, obstacles, facilitators and advantages without looking for a special topic of implementation. We also searched literature describing the transition from onsite to online teaching, before and after the pandemic. We performed the search in the electronic databases PubMed, EBSCOHOST (CINHAL plus; Education research Complete); Google (Google Scholar); EMBASE (Ovid). For RQ1, we included studies evaluating the transition from onsite to online either blended or a full transition. For RQ2 studies describing medical faculties’ experiences with DLT during the pandemic was the primary focus. We included quantitative, qualitative, and mixed methods studies.

In the second step, the search results were downloaded to EndNote® desktop software and screened according to relevance for RQ1 and RQ2. Empirical studies published in peer-review journals meeting review-specific eligibility criteria were included. Studies that did not include medical faculty experiences were excluded.

In the third step, one of the authors extracted the data. The full text of included papers was uploaded to NVivo 12 computer software [38]. Thematic analysis was used to organize data in a structured evidence-based approach [39, 40]. The upper level deductive themes, identified from the initial literature review were applied for initial extraction: requirements, obstacles facilitators and advantages.

In the fourth step, we identified subthemes from the selected articles. Following the logical order of the thematic analysis framework, codes were identified and decided with multiple researchers.

### Interviews

To investigate the RQ3, semi-structured interviews with curriculum managers were implemented. The steps to collect data and to analyse the interviews are described below.

The development of the question route based on the thematic synthesis of the reviewed studies published before the pandemic, the question route was developed according to the procedure suggested by Gideon [41], and high-level themes of relevance to the study were identified. For each of the identified main themes the authors wrote two questions each. Four questions per theme were validated by two authors and the two best fitting were selected by consensus. The resulting questions were revised in two steps, first, through a critically semantic review of ambiguous wording or meanings. Second, 2 pilot interviews were conducted to assess the level of understanding of the presented questions. The pilot offered the possibility to discuss with targeted participants and to refine the question route according to the collated feedback. The resulting question route is shown in the Supplementary Material 1: Appendix II.

#### Participants

A purposive sampling method was applied and key persons from 7 Swiss Medical Schools (Basel, Bern, Freiburg, Geneva, Lausanne, Zürich, and the Swiss Federal Institute of Technology in Zürich) were invited. At two institutions, more than one person participated in the interview, resulting in a total of eleven interviewees (10 Male; 1 female, 10 physicians and 1 natural scientist, 5 Vice Deans; 6 heads of curriculum management). ‘Curriculum mangers’ hold crucial stakeholder positions within the Swiss educational system and are typically entrusted to one or two individuals per school. Their tasks involve deep insights into operative issues at each University. Using interviews, we could capture each participants’ unique experiences. At Swiss Universities, undergraduate medical education is a six-year programme and is based on the Bologna reform, with a Bachelor and a Master programme, each covering 3 years of study. Students acquire knowledge in medical basic sciences during the bachelor study, combined with early clinical experiences. The master-degree includes clinical teaching and experiences in all the main medical disciplines. All the medical schools apply the same national learning objectives framework [42].

#### Procedure and setting

The interviewees were recruited by e-mail and received information on the purpose and procedure of the interviews. Full anonymity for data analysis and results were guaranteed. Interviewees participated by Zoom (*N* = 6) and in person (*N* = 4), between 18 May and 16 July 2020. All interviews were conducted by the same interviewer in English, following the question route shown in Table 1.

**Table 1:** Question route

Section	Questions
**Institution and Tasks of Interviewee**	Can you describe the tasks of the unit/institution where you work within the faculty of ? What are your personal roles/tasks within your institution or unit? Do you teach? If yes, did you also teach during the lockdown? If yes, what were your experiences? Do you or other lecturers have experience with online teaching? If yes, for how long and which subjects?
*Organization and Management of Teaching Activities: Before, during, and after the lockdown: General experience in managing the situation*	
**Immediate Implications**	What were the immediate practical implications of the COVID-19 crisis for university lecturers and teachers in your institution? Which regular teaching activities (Lectures/Courses/Bedside teaching/others) were affected? Approximately, how many teaching hours per week were affected? How long was the transition to online teaching (weeks/days)? What was the most challenging aspect of this transition? Can you mention some reactions from teachers you know of?
**Curriculum Adaptations: Before/During/After COVID19**	How much teaching was conducted online before the lockdown (estimated percentage)? Which parts of teaching are offered online during the lockdown? Which parts cannot be offered online during the lockdown (challenges and solutions)? Which parts are not replaced with online teaching and are instead in the students' own learning responsibility? What will change when on-site teaching restarts?
**Institutional Factors**	Does your institution have a quality concept or criteria for online teaching? What support does your institution offer to lecturers for optimizing online teaching (technical and didactical)? What do you know about lecturers' reactions to their new way of teaching? What needs and suggestions for improvements were reported? Positive experiences or challenges?
**Technology Adaptations**	What learning management system do you use at your institution (Ilias, Moodle, Olat, etc.)? What technical tools were used for online teaching before the lockdown? Did you or the lecturers experience any technical problems? How were they resolved?
**Teaching Methods and Activities: Before, During, and After the Lockdown**	Did your institution have a strategic plan for online teaching and learning before the current situation? What new teaching methods were implemented due to the current situation? What would you do differently another time, having to switch to online teaching immediately? Has your institution succeeded in teaching practical knowledge online?
**Online Learning Opportunities**	What online learning options/platform subscriptions are offered at your faculty (e.g., Amboss)?
**Assessment/Assignments Adaptations**	How did COVID-19 affect the implementation of exams? Do you align your exams with the new online teaching methods during this time?
*General Issues*	
**Community**	To what degree do lecturers and teachers maintain contact with their colleagues during the lockdown?
**Cooperation**	Are you in contact with other medical faculties during the lockdown to exchange experiences related to online teaching?
**Final Questions**	What have you personally learned from this experience?

Interviews lasted between 50 and 70 min and were audio-recorded. The structure of the question route was followed but spontaneous information and associations evoked by the questions were explicitly encouraged. The data were transcribed verbatim. After transcription, the interviewees approved the transcript and confirmed that their responses could be included in the analysis.

### Analyses

We analysed the interview data using NVivo. The question route provided a first draft of deductive themes. In addition, we identified inductive themes through a thorough review of repeated concepts. To address RQ 3, we performed a comparative analysis between the results from the literature addressing pandemic experiences and the interviews.

We followed the method for comparative thematic analysis, purposed by Riggs [43] to gain a better understanding of the institutions’ experiences during the pandemic in relation to emerged themes from the literature. This comparative analysis was performed using NVivo. Categories were formulated based on the themes analysed in NVivo. Two reviewers decided on the categories with 100% interrater reliability.

## Results

### Literature review

A total of 1643 potentially relevant titles and abstracts were screened. 24 articles met the eligibility criteria and were included for full text screening. Of the included articles, 15 reported on DLT more general and nine articles reported on DLT during the Pandemic. Four of the included papers were comprehensive reviews which offered an overview of enablers and barriers to DLT in health education [5, 15, 44, 45].

### Synthesis of DLT literature on DLT experience before the pandemic (RQ1)

The thematic analysis of the literature confirmed four overall themes, requirements, obstacles, facilitators and advantages (Table 2).

**Table 2:** Summary of results from the literature published before the pandemic

Institution	Teachers	Students
**Requirements**	**Requirements**	**Requirements**
- Institutional structures [5] - Implementation strategies [5, 15] - Training for communication and interaction with students [45]	- Time to prepare the materials [17]	- Time, social interaction [46] - Infrastructure, support [46] - Technical and academic skills [47]
**Obstacles**	**Obstacles**	**Obstacles**
- Not suitable for all learning modalities [15] - Lacking well-established onsite teaching culture [15]	- Difficulty to implement online interactions with students [18] - Not aware of new teaching roles, demands, competencies [16, 17, 47] - Not aware of new technological possibilities and developments [16] - Lack of resources to change [16] - Unaware of students’ learning [16] - Low technical skills [5, 15, 17] - Technology avoidance [15] - Insufficient communication and support from institution [5]	- Administrative issues [46] - Technical problems [46] - Costs, access to the Internet [46] - Confidence to learn online [46] - Resource-intensive, too many choices [15] - Poor motivation [15]
**Facilitators**	**Facilitators**	**Facilitators**
- Better administration [14] - Cost-effectiveness [14] - Less student/lecturer time [14]	- Greater flexibility as to where to work from [14] - Aids to improve teaching [7, 15] - Fosters role as a coach [48, 49]	- Facilitate learning [15] - Aided transfer to practice [15] - Systematic way of learning [15] - Enhancing active learning [7, 15] - Personalized learning [9] - Foster self-directed learning [10, 11]
**Advantages**	**Advantages**	**Advantages**
- Increasing the quality and effectiveness of education [12–14] - Ease of standardization and keeping content up to date [12–14]	- Transparency and accountability [12–14]	- Better information accessibility [12–14]

### Synthesis of the DLT literature addressing the situation during the early pandemic-phase (RQ2)

The second part of the literature review was used to add to and support the analysis of the interviews. The same primary themes were identified (Table 3).

**Table 3:** Summary of results from the literature reporting pandemic related experiences

Institution	Teachers	Students
**Requirements**	**Requirements**	**Requirements**
- Faculty training [19, 50] - Communication between key players [19, 50] - Time management [19, 50]	- time [19, 50] - support in implementing online assessments [19, 50] - Communication: institution and students [19, 50]	- learner guidance, instructional support, adjustment to DLT [21, 25] - Feedback in the learning process [21]
**Obstacles**	**Obstacles**	**Obstacles**
- Delivering practical courses [51]	- Lack of faculty training and institutional support [19, 50]	- Low acceptance of online lectures [21] - Feeling of isolation, lack of interaction with teachers and peers [20, 21, 25] - Lack of technical infrastructure [25] - Internet and technical problems [52]
**Facilitators**	**Facilitators**	**Facilitators**
Cost-effectiveness [25]	**–-**	- Self-efficacy [53] - Enjoyment [53, 54] - More learning flexibility [53]
**Advantages**	**Advantage**:	**Advantages:**
- increased confidence in DLT effectiveness [19, 50]	- gained experience with online learning and teaching [19, 50]	–-

### Synthesis of the interviews (RQ3)

Curriculum managers shared their perspectives during interviews that institutions were relatively un-prepared for the quick transition from onsite to online at the onset of the pandemic (Table 4). Given that interviewees consisted of curriculum managers only, the presented themes of teachers’ and student’s are presented from the perspective of curriculum managers and from their own experiences as teachers.

**Table 4:** Summary of results from the interviews

Curriculum managers	Teachers from the perspective of curriculum managers	Students from the perspective of curriculum managers
**Requirements**	**Requirements**	**Requirements**
- implement a DLT structure -develop the needed DLT strategy - set the standards for quality in DLT	- training for online teaching and technical training—more time for DLT implementation	- more guidance regarding learning
**Obstacles**	**Obstacles**	**Obstacles**
- administration issues - communication issues	- Lack of the right attitude and motivation to implement and use technology for DLT	- lack of guidance on how to learn the materials—lack of group teachings - lack of practical teachings
**Advantages**	**Advantages**	**Advantages**
- new tools for DLT implementation were discovered -gained experience on how to teach online	-gained experience on how to teach online	–-
**Facilitators**	**Facilitators**	**Facilitators**
-working with highly competent people -having high technological equipment -good sense of collaboration between universities	-positive attitude to handle the situation -good collaboration between teachers	- flexibility to learn at your own pace and place -positive attitude to handle the situation

### Comparative analysis between literature and interviews: Common aspects.

**Fig. 1:**
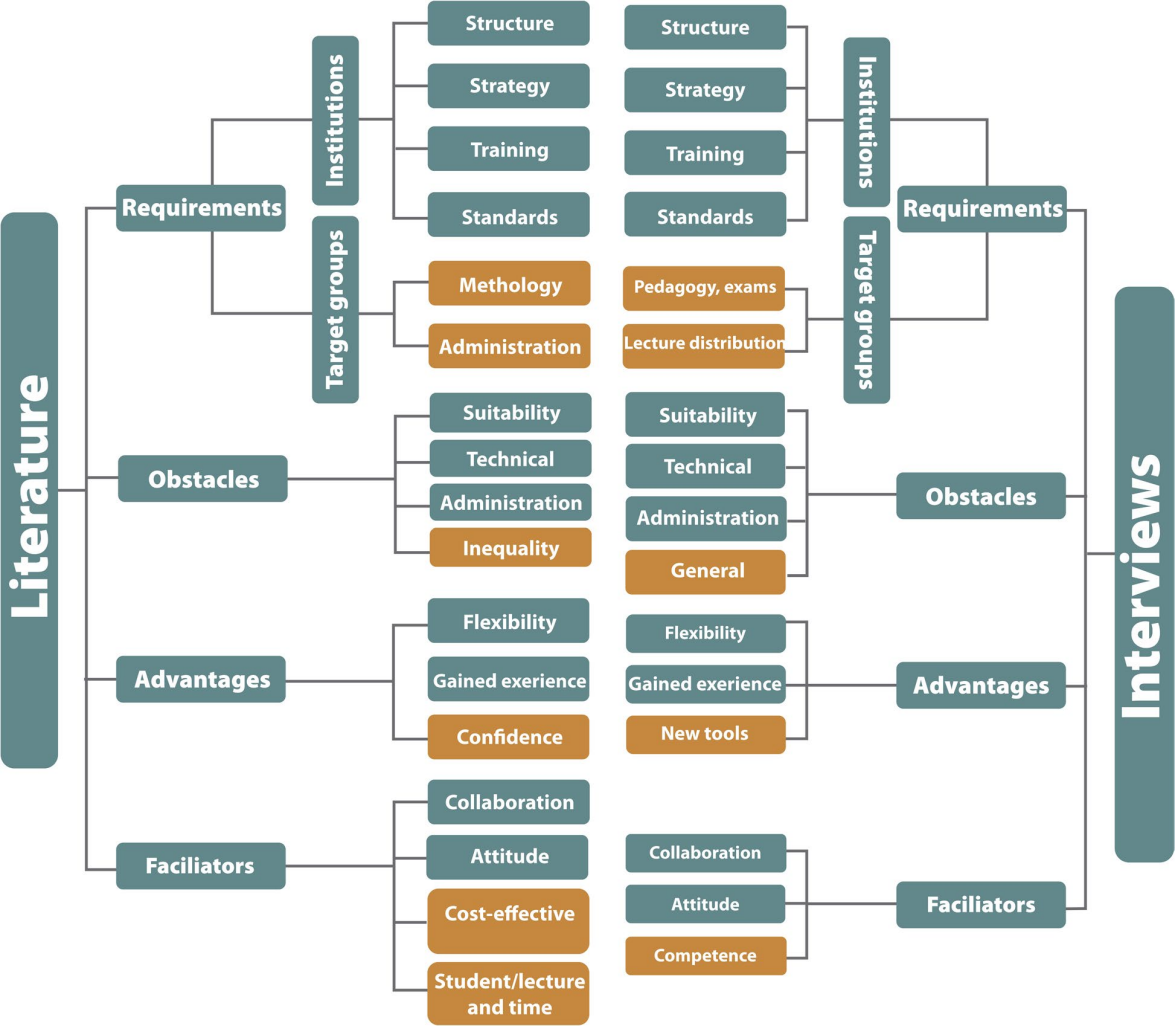
Common (dark) and different elements (light) between literature and interview findings addressing DLT experience

Figure 1 illustrates the results from the integrated thematic analysis and highlights commonalities (dark) and differences (light) between literature and interview findings addressing DLT experience.

#### Requirements

We noticed a lack of standards regarding the quality of the online teaching is apparent. Interviewees suggested that it is not necessary to focus on the quality of teaching in times of crisis, although a need for best practice guidelines for DLT was stated. ‘Structure’ and ‘Strategy’ were selected as relevant themes particularly for students, as they can reduce stress and anxiety to manage assigned tasks. (Abbreviation for quotes below: L = from literature; I = from the interviews).

L: “Providing structure was a key element of effective support for managing stress and anxiety. (p 292 [55])” (Structure).

I: “…that was for students maybe the hardest point, that a structure was crushed” …. The teaching strategy was called into question, exposing that the reluctance towards transition to online teaching my not be the relevant issue, instead there is a need that educators rethink teaching and clarify the purpose of teaching. (Structure).

L: “Given the limited time available for transition to remote/online delivery, the authors experiences demonstrated an almost consistent strategy of repurposing existing material (page 294 [55])”. (Strategy).

I: “…we should think about the teaching strategy in general and not only the online teaching strategy”. (Strategy).

The need for teacher ‘training’ is a recurring issue. Due to the immediate transition from onsite to online training, no support could be offered to the teachers at the rate required. From our findings, we present unique means of training support.

L: “When tasks are beyond their training, additional training must be provided… (page 13 [42])” (Training).

I: “Teachers had personal coaching, they were able to make a date with us to come to our house and get supported to record and upload their lecturers online.” (Training).

#### Obstacles

One of the most frequent obstacles was a lack of ‘technical’ knowledge, particularly for the teachers, which was a direct consequence of the reluctance to adapt, combined with minimal institutional support and lacking institutional structures.

L: “A common challenge reflected on was that of information technology (IT) skills, and the lack of prior training or knowledge for effective online education delivery practices. (p 289 [55]). (Technical).

I: “We had to choose the right tool for keeping up the pedagogical concepts as much as we could.” (Technical).

#### Advantages

DLT offers ‘flexibility’ of temporal teaching-delivery, as indicated in Fig. 1. Another advantage and effect of the pandemic was gained DLT experience, which indeed can facilitate DLT implementation. When users overcome resistance due to unknown or unfamiliar territory, ‘new experiences’ on a learning platform could be further encouraged.

L: “The experience respondents have had during the first few weeks of COVID-19 has increased their confidence in the effectiveness of online medical education. (page 9 [56])” (new experience).

I: “So, the students could take this class and then that class in the order they wanted, and in the rhythm wanted, or could stop and look for more material.” (Flexibility).

#### Facilitators

Both the literature and interviewees stress that a positive attitude towards implementing and using DLT possibilities is a prerequisite for success (See Fig. 1). ‘Collaboration’ was one of the most mentioned elements to assure a higher acceptance of DLT. In the interviews, the importance of communication was stressed between teachers and between universities.

L: “Collaboration or interaction between learners and facilitators would influence an attitude of sharing knowledge, which is one of the crucial elements of e-learning’s shared enterprise.” (p 8 [15]] (Collaboration).

I: “I also experience that there is a good sense of teamwork with the whole faculty in an extreme situation.” (Collaboration).

### Comparative analysis between literature and interviews: Deviating aspects

Most differences between the literature review and interviews were found related to ‘Requirements’ of stakeholders (students and teachers). No deviating insights between the literature and interviews on requirements at the institutional level were noted. Also, we found relevant differences between the literature and interviews under characteristics of ‘Obstacles’.

#### Requirements of teachers and students

The literature reports requirements for implementation of DLT including student guidance, time and training. The interviews, however, offered a richer picture of requirements. In the process of course delivery and digital uploads, participants noted a positive experience of interacting with their ‘institution’. At some institutions, however, this process required more time than planned, due to a lack of prior experience and ‘strategy’ with DLT. Delivery of online ‘exams’ were challenging due to the possibility of cheating.

I: “I would say that it was good to see that in this modern crisis, our system was able to adapt very quickly and very effectively.” (Pedagogy, exams).

I: “So here it took maybe double that time < as planned > .” (Lecture distribution).

I: “There was no strategic plan for online teaching before.” (Pedagogy, exams).

I: “We reduced the time of the exam to minimize the possibilities for collaboration.” (Pedagogy, exams).

I: “It's a wonderful opportunity to increase the part of blended learning in our curriculum.” (Lecture distribution).

#### Obstacles

In published research, ‘inequality’, in terms of access to resources, persist as a recurrent theme. This did not emerge from the interviews, which is likely to be an effect of high socioeconomic standards in the Swiss educational system among all stakeholders. However, the interviewees mentioned a variety of other Swiss obstacles influencing students’ learning and teachers’ coping (theme ‘general’). More specifically, students were overwhelmed with the amount of available information. A lack of interaction between teachers and the students was reported in the interviews in many different ways.

I: “The students prefer to have as few different sources as possible.” (General).

I: “Students would have preferred to stay in presence at the university.” (General).

I: “There was less discussion between the students and less interaction with teachers and students.” (General).

I: “Of course, interactivity was an issue. And so, we learned to use the chat room for asking questions, and to break the zoom call into smaller groups for student presentations.” (General).

#### Facilitators

As shown in Fig. 1, also for the theme ‘facilitators’, the insights gained from the interviews were richer than the literature. The subtheme ‘cost-effectiveness’ was only mentioned in the literature.

L: “…the studies included addressed the cost-effectiveness of eLearning versus traditional learning. (page 11 [25])” (Cost-effectiveness).

In the interviews, ‘competence’ emerged as a new theme. The presence of highly skilled persons at the university facilitated the DLT transition.

I: “What I learned is that we have wonderful people around us and they have competence that sometimes they don't need to use that much. And all of a sudden, because of the crisis, you see how competent they are in some fields, how willing and involved they are to help and make things happen.” (Competence).

#### Advantages

In the literature the theme Increased ‘confidence’ in new technologies due to the pandemic was unique (28). In the interview a similar but not identical subtheme emerged: discovery of ‘new tools’, meaning that the forced online teaching brought teachers a chance to try new tools.

L: “…COVID-19 has boosted their < teachers > confidence in the effectiveness of online education…(page 1 [56])”. (Confidence).

I: “So personally, I found it was a very big opportunity to discover some of those tools.” (New tools).

## Discussion

In this study, we capture the experiences of the rapid transition to online teaching in the pandemic from literature and Swiss curriculum developers. The comparison between views of curriculum managers across Switzerland with findings from international literature brought important insights and served to view the Swiss experience in a broader context. In the following we reflect how the findings provide answers to our three research questions.

The first research question investigated salient factors that influence the success of DLT activities. Through the thematic analysis of the relevant literature, we could identify four main themes: ‘requirements’, ‘obstacles’, ‘advantages’, and ‘facilitators’, which showed to be applicable also for the interviews. We developed the question route for the interviews based on this literature and were thereby able to identify further salient deductive themes. Through analysis of the selected studies, a structure of related subthemes was extracted providing a framework for further analysis (Fig. 1). Although a need for more DLT was already known prior to the pandemic, our findings suggest that the former reported lag in DLT implementation was most likely caused by ignorance related to the known obstacles at the institutional level.

The second research question relates to early international experiences of implementing DLT initiatives during the pandemic in medical education. The pandemic led to a landslide of DLT developments and ad hoc solutions to transition from onsite to online. A comparison of the new experiences with earlier literature clearly shows that obstacles had to be addressed through mandatory initiatives at an institutional level due to the crisis. The new focus changed from supporting an onsite teaching culture to active practical implemention of DLT.

Delivering of clinical training to learners was critically reduced and could only partially be delivered online. Innovative digital approaches further facilitated delivery of hands-on teaching by Incorporation virtual-reality applications or 3D anatomy software with a decrease in cadaver dissection [57]. This may be applied to other fields in the future.

Our results show a differentiated picture of requirements, obstacles and facilitators for DLT implementation. We note that several challenges that emerged from our interviewees are also reported in recent publications, indicating that early challenges are not a pandemic problem easily solved. These include economic repercussions of the pandemic; social distancing affecting the delivery of medical education, the surge of patients affecting redeployment of personnel and potential compromises in core training; and the overall impact on the wellness and mental health of trainees and educators [58]. Post pandemic, current literature continues to stress that early noted obstacles must be addressed in the future [59].

One recent review shows that readiness to respond to the situation by the institutions was a facilitator for success. The remaining barriers are the lack of planning, –resources, and –interactivity between teachers and students [59]. Other findings from the pandemic point to the same direction: attitude to e-learning, networking and interdisciplinary collaborations affected the implementation of DLT. Challenges like inadequate interactions, time constrictions, and administrative issues will continue to be obstacles in any situation demanding a quick move to online learning in the future [60]. Therefore, it continues to be important to develop the necessary infrastructure and assure the adequate resources [28]. Also, the need to be more specific about how we teach has been noted in recent literature [60].

The third research question address the Swiss experience. Our analysis shows commonalities and differences between published research and the perspective of curriculum managers. The interviews identified specific themes that are also relevant for the continued implementation and management of DLT. Within the theme of ‘requirements’ further differences were noted for obstacles, facilitators and advantages, which we will present in more detail.

The interviews revealed that the Swiss faculties were mostly unprepared for the complete shift to DLT in the first lock-down. The crisis management, as reported from the interviewees, exposed important requirements related to structures, strategy, standards and implementation, directly impacting administration and communication.

Another barrier revealed by the interviewees included quality assurance issues. This finding is consistent with an earlier study [61], in which the authors concluded that one of the most imperative means for implementing online modules is encouraging collaboration among all departments and stakeholders. An organized and clear institutional approach is required to formulate a well-regulated and efficient system which can facilitate the adoption of useful methodologies by faculty members for implementing an online learning [61]. Specifically, the interviewees raised concerns on how to sustain the high quality of Swiss exams when they are delivered online. Existing Swiss medical exams are normally not designed for open book solutions, and so in order to reduce fraud, exam time was reduced to a minimum, limiting students time to investigate answers from online sources. This concern was not noted in this way from our findings within the literature.

The provision of support delivered with access to ‘help desk’ or a mentor is essential to effective teaching online. Institutions were faced with the challenge to radically increase and rapidly scale up online support for DLT from the onset of the Covid-19 pandemic. This was a reported challenge from all Swiss faculties, independent of the former DLT progress. Consequently, both new literature and our interviews show that foci shifted from planning to doing. These insights are also emphasized in recent literature discussing DLT during the Pandemic [62, 63]. Experiences related to DLT during the pandemic uncover new obstacles including a lack of institutional support for faculty training, a decrease in student engagement and challenges in implementing assessments online, insufficient communication between the stakeholders, and time management, as well as a host of additional challenges [19, 20, 29–31, 50, 56, 64–67]. The delivery of most practical skills-based training was not possible. Some medical disciplines had to take extensive effort to adapt to the DLT setting (e.g. surgery, internal medicine), as teachers and students need opportunities for safe exercise opportunities or demonstrative learning, communication, and group dynamics [68]. However, online learning can serve as an efficient resource for clinical practice if the method is upgraded through the integration of modalities such as virtual simulation technologies and computer-based models of real-life processes [69]. Further, online learning for clinical skills can serve as an excellent preparation for onsite clinical training, e.g. for communication training [70, 71]. Our findings in this research project are congruent with the literature that communication training online was feasible and surprisingly well accepted [72]. For the future, given that online training is well implemented, it can produce multiple benefits for clinical learners by providing controlled opportunities to practice rare and critical events in safe environments excluding the risk to patients [73].

According to the interviewees, many teachers show general enthusiasm for DLT. However, as the traditional teaching is their main source of experience—they simply lacked technical knowledge and DLT experience resulting in initial scepticism towards DLT. Initially, technical knowledge was a hinderance for learners, but this evolved into positive encounters through accumulated experience. At the start of the pandemic the teaching format remained unchanged as all the lectures were uploaded as podcasts. So, the issue was not so much to achieve quality, but only basic online teaching, without optimisations or innovations. Due to lack of experience, training and time, even this simple aim was a challenge, as teachers were not trained or prepared to deliver online content. One of the most common barriers was technical insufficiency, including deficits in educators’ basic computer skills. The themes identified in our study complement previous studies [74].

One of the main aspects discussed in the interviews was that teaching quality is not dependent on the mode of delivery; online or onsite. There is a relevant distinction to be made between the quality of teaching and teaching method: “*It’s actually quite depressing the quality of teaching sometimes, but it’s not about online but interactivity. It’s not that because you do online, you have to be more interactive.*” The most poignant reflection from our study is the consistently reported lack of good quality teaching. Does online teaching demand its own didactic, or does it make sense to focus on the holistic view of currently teaching? Newer studies also stress this question [42]. We could document that making the first step is important, a major success factor was that earlier resistances from the teachers had less impact, than expected. This enabled all stakeholders to gain new experiences and trust in their own digital competences.

The interviewees noted that students in general experienced a lack of guidance, deficits in interaction with their teachers, as well as lacking optimised solutions for clinical demonstrations, lack of direct contact, spontaneous interaction and clarifications of their questions. The known deficits of online learning, captured from the literature [20, 21, 25] about students’ feelings of isolation resulting in reduced mental health, were not explicitly noted by the interviewees. Reflecting on this point, our participants took part in interviews at the beginning of the Covid-19 pandemic, where students’ ‘isolation’ may not have yet had significant impact.

### Critical reflections

This study reports on experiences of curriculum managers at the start of the pandemic, where the initial adaptation to DLT was still new. The relevance of this study would be reduced, if experiences changed during the pandemic and different issues became dominant at a later stage. Our impression from the newer literature, reported above, is that many challenges, as reported in the Tables 1, 2 and 3, remain. Long-term development of DLT is needed to address all requirements, obstacles, advantages and facilitators related to successful implementation.

The interviews were conducted in English, and consequently there may be a potential bias introduced here as subtle nuances might be lost when non-native speakers express complex ideas in another language. However, all interviewees were fluent in English, and approved the transcript. The use of English facilitated the use of quotations without misinterpretation from translation.

We interviewed 11 curriculum managers from 7 Swiss medical Schools, which may be considered a small sample raising questions of the representativeness information gathered. However, this sample represents important stakeholder roles within the Swiss educational system and are normally in the responsibility of one to two persons. In this sense, the sample is representable. We also investigate perspectives of students and teachers- as reported by the interviewees, which may not capture the true perspective from these groups at large. However, data from the interviews is validated as many of the emergent themes from the interviews are well aligned to the literature. Our findings illustrate a high degree of converging experiences, indicating saturation of data. Variations between the institutions were noted, some reporting smoother DLT integration than others.

In reflecting on our findings, it is evident that educators require a diverse skill set to effectively support digital teaching initiatives, encompassing online communication, time management, technical proficiency and a transition to a more facilitative role.

## Conclusions

Since the onset of the pandemic, education systems have unexpectedly transitioned to a new era of education with more focus on DLT. This study underscores the essential role of strategic and operative support of DLT at an organisational level, taking the needs of students and teachers into consideration.

A strategic focus for DLT is needed including reflections on the future goals of teaching in medical education. Educators must be enabled to support students in effective and efficient learning, in multiple ways [73]. Medical schools and education institutions are advised to promote facilitating factors to effective online learning and address barriers hindering effective DLT implementation. For an optimal implementation of DLT, our findings support an alignment between key players’ requirements. The need for alignment is evident in the literature for all three central stakeholders, institutions, teachers, and students. With facilities and resources available to Swiss medical students, it might be expected that education institutions could offer and implement high quality DLT with relative ease. Our results show this was not the case during the pandemic. Sustained financial support for a continued DLT development together with a clear strategy based on didactic insights is needed.

Our research reports a lack of institutional structures, communication, digital literacy, teaching strategies, as well as a theoretical foundation for DLT implementation. A conceptual DLT framework adapted to the Swiss universities is needed to prepare for a period of transition to long-term sustainable solutions. Findings have the potential to advise medical institutions in how to implement DLT in a didactical sustainable way, to provide high quality online teaching post pandemic. Changing a system takes time. After all the experiences during the pandemic, for good and for bad, reaching the true potential of DLT will be a journey.

While our research focuses on the challenges and strategies within medical education's shift to DLT, the identified requirementfor institutional structures, clear communication, and a robust theoretical foundation for DLT is universally applicable across disciplines. The journey to fully realize the potential of DLT, underscored by our findings, offers valuable insights for all educational institutions striving for sustainable, high-quality online teaching in a post-pandemic world.

### Supplementary Information

**Supplementary Material 1.**

## Data Availability

The datasets generated and/or analyzed during the current study are currently not publicly available because these are interviews from well-known heads of curriculum management and vice deans. We have a written declaration that we will ensure their privacy. Availability of source data can be provided only on reasonable request and the interviews will be modified to ensure absolute anonymity. I this case please contact the first author.
