# Supplementary material for: Swiss medical schools’ experiences with online teaching during the COVID-19 pandemic in light of international experiences
Source: BMC Med Educ. 2024 Mar 6;24:242. doi: 10.1186/s12909-024-05218-3 (PMC10916260; doi:10.1186/s12909-024-05218-3)
Supplement: Supplementary file 1 — Supplementary Material 1. [file 12909_2024_5218_MOESM1_ESM.pdf]

APPENDIX I

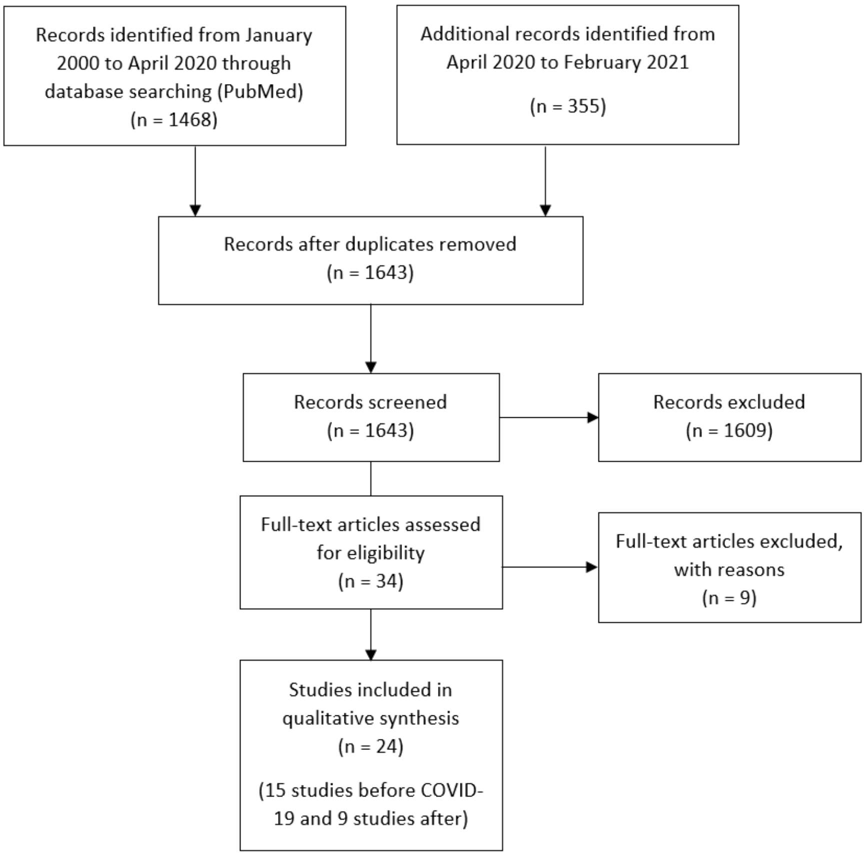

Overview of literature found and integrated for both lines of search (before and during the pandemic): We included studies of any methodology dating from 2000 to 2021 that evaluated the experiences of institutions, teachers and students with online learning and teaching. Exclusion criteria: We included only studies published in English and excluded studies which did not focused on the experiences of the above-mentioned stakeholders, as well as studies which did not follow an empirical point of view. As such all studies evaluating theoretical considerations were excluded. PICO: Population-- institutions, teachers and students / Intervention—DLT / Comparison—before and after COVID / Outcome – the experience of implementation of DLT.

The search code:

("online learning"[Title]) OR "online education"[Title]) OR "digital learning"[Title]) OR "digital education"[Title/Abstract])) OR "blended learning"[Title]) OR "blended education"[Title]) OR "blended teaching"[Title]) OR "online teaching"[Title]) OR "digital teaching"[Title])) OR "distance learning"[Title]) OR "distance teaching"[Title]) OR "distance education"[Title]

APPENDIX II

Question route for the interviews

**Main topics implemented in the question route and example of questions**
**Requirements**
-Did your Institution have a strategic plan (regarding didactical methods) for online teaching and learning before the current situation?
What kind of support does your institution offer to the teachers in order to enable them to optimize their online teaching?
**Needs**
Which unit in your organization offers technical support?
Which unit in your organization offers didactical support?
**Obstacles**
What kind of regular teaching activities (Lecture / Courses / Bedside teaching) was affected?
Did you experience technical problems?
**Advantages / Needs**
What have you personally learned from this experience?
What do you know about teachers and students'reactions to their new way of teaching?
**Facilitators**
Which aspects, if any, made the new sitation manageable for you?
Which aspects, if any, made the situation manageable for the teachers and students?
**Additional questions**
Do you know to which degree the teachers keep up contacts with their lecturing colleagues during the lockdown time?
Were you in contact with the other medical faculties during the COVID-19 pandemic in order to exchange experiences related to online Teaching?
